# Involvement of Akt/CREB signaling pathways in the protective effect of EPA against interleukin-1β-induced cytotoxicity and BDNF down-regulation in cultured rat hippocampal neurons

**DOI:** 10.1186/s12868-018-0455-7

**Published:** 2018-09-06

**Authors:** YiLong Dong, KangJing Pu, WenJing Duan, HuiCheng Chen, LiXing Chen, YanMei Wang

**Affiliations:** 1 School of Medicine, Yunnan University, 2 Cuihu Bei Road, Kunming, 650091 Yunnan People’s Republic of China; 2 The First Affiliated Hospital of Kunming Medical University, 295 Xichang Road, Kunming, 650031 Yunnan People’s Republic of China

**Keywords:** Eicosapentaenoic acid, Brain-derived neurotrophic factor, Akt, Interleukin-1β

## Abstract

**Background:**

Our published data have indicated that the omega-3 polyunsaturated fatty acid eicosapentaenoic acid (EPA) provides beneficial effects by attenuating neuronal damage induced by interleukin-1β (IL-1β), and up-regulation of the expression of brain-derived neurotrophic factor (BDNF) represents a crucial part in the neuroprotective effect of EPA. However, the mechanisms of how EPA regulates BDNF expression remains incompletely understood. The present study investigated the role of Akt/CREB signaling in the effect of EPA on BDNF expression and its neuroprotective effect.

**Results:**

The present results showed that IL-1β reduced hippocampal neuronal viability and that EPA showed a concentration-dependent neuroprotective effect, but the neuroprotective effects of EPA were abolished by inhibition of Akt using KRX-0401, an inhibitor of Akt. Treatment of hippocampal neurons with EPA also ameliorated the decrease in Akt and CREB phosphorylation induced by IL-1β and BDNF down-regulation mediated by IL-1β. However, inhibition of Akt reversed the effect of EPA on levels of p-Akt, p-CREB, and BDNF.

**Conclusions:**

Our data indicate that EPA elicited neuroprotection toward IL-1β-induced cell damage and BDNF decrease and that its effects potentially occurred via the Akt/CREB signaling pathway.

## Background

Although the pathogenesis of neurodegenerative diseases, such as Alzheimer’s disease (AD) and Parkinson’s disease (PD), remains to be elucidated, neuroinflammation is considered one of the underlying factors of these disorders [1, 2]. Inflammation occurs in vulnerable brain regions of individuals with neurodegenerative disease, including the cortex, striatum and hippocampus, which is characterized by microglia activation and abnormally elevated levels of proinflammatory cytokines, such as interleukin-1β (IL-1β), IL-6 and tumor necrosis factor-alpha (TNF-α) [3]. The excessive proinflammatory factors may cause persistent neuronal damage through different signaling pathways, including suppression of brain-derived neurotrophic factor (BDNF) [4]. BDNF is a critical neurotrophin associated with neuronal survival, differentiation and synaptic plasticity [5]. Accumulating studies have demonstrated the link between low BDNF levels and increased neuronal injury during a neurodegenerative disorder [6, 7]. Therefore, BDNF-augmenting treatments shall be beneficial to rescue neurons from neurodegenerative disease.

Eicosapentaenoic acid (EPA) is an omega-3 polyunsaturated fatty acid that cannot be synthesized by the human body. Our main sources of EPA are cold-water fish. There is increasing scientific evidence from epidemiology and animal studies linking EPA intake with brain health, which demonstrated parallel alterations between diets high in EPA and reduced risk of neurodegenerative disease [8–12]. Previously, we have reported that EPA markedly attenuated the IL-1β-induced BDNF decrease in the hippocampus, which may provide beneficial effects against inflammation-associated neurodegenerative changes [13]. However, the mechanisms underlying how EPA modulates BDNF still remain unclear.

Previous studies have shown that cAMP-response element binding protein (CREB) acts as a transcription factor and is present in many types of neurons [14–16]. Importantly, CREB-binding sequences have been identified in the BDNF gene [17]. CREB may be located upstream of BDNF and may up-regulate the expression of BDNF [18]. Before it works, some protein kinases, such as protein kinase A (PKA), Akt (also known as protein kinase B) or mitogen-activated protein kinase 2, are required to phosphorylate CREB at serine-133 and convert CREB to its active form [19]. Recently, since research has suggested that EPA can modify the activity of several protein kinases, including Akt [20], we therefore hypothesized that EPA would protect neurons by rescuing low levels of BDNF from IL-1β toxicity by regulating the Akt/CREB signaling pathways. In the present study, using the IL-1β-induced neuronal degeneration model, we investigated the protective role of EPA against cellular toxicity and examined the potential involvement of the Akt/CREB pathway in EPA-mediated neuroprotection and BDNF regulation.

## Methods

### Materials

IL-1β was purchased from R&D Systems (Minneapolis, MN, USA). 3-(4,5-Dimethylthiazol-2-yl)-2,5-dimethyltetrazolium bromide (MTT), DNAase1 and 1-β-D-arabinofuranosylcytosine were obtained from Sigma-Aldrich (Saint Louis, MO, USA). Neurobasal medium, B27 supplement, trypsin and TRIzol were purchased from Invitrogen Corporation (Carlsbad, CA, USA). The protease inhibitor mixture, phosphatase inhibitor, BCA protein assay kit and enhanced chemiluminescence substrate kit were obtained from Pierce Biotechnology (Rockford, IL, USA). RIPA lysis buffer was purchased from Beyotime Biotechnology (Shanghai, China). Akt (catalog No. 9272), phospho-Akt (catalog No. 5012S, Ser473), CREB (catalog No. 4820) and phospho-CREB (catalog No. 9198, Ser133) antibody were purchased from Cell Signaling Technology (Danvers, MA, USA). BDNF (catalog No. ab108319) and GAPDH (catalog No. ab37168) antibody were obtained from Abcam (Abcam, England). KRX-0401 (catalog No. S1037) was purchased from Selleck Chemicals LLC (Houston, TX, USA). An Ominiscript RT Kit and SYBR Green Kit were purchased from QIAGEN (Hilden, Germany). All other chemicals used were of the highest grade commercially available.

### Cell culture

The primary hippocampal neurons were prepared as previously described with minor alterations [21]. Briefly, hippocampal tissues from Sprague–Dawley rats at embryonic day 18 were dissected in cold Ca^2+^ and Mg^2+^-free Hank’s balanced salt solution (HBSS), the tissues were mechanical dissociation using a Pasteur pipette after incubation with 0.25% trypsin and 50 µg/mL DNAase1 at 37 °C for 10 min. The cell suspensions were then transferred to a new tube, centrifuged at 1200 rpm for 3 min. The cell pellet was resuspended in serum-free neurobasal medium supplemented with 2% B27 supplement, 100 U/mL penicillin, and 100 µg/mL streptomycin, and then seeded on cell culture plates pre-coated with poly-l-lysine-coated. The cultures were grown in a humidified 5% CO_2_ atmosphere at 37 °C. 1-β-D-arabinofuranosylcytosine was added to the neuronal cultures on day in vitro 3 (DIV 3) to a final concentration of 2 µM to reduce glial cells proliferate. All treatments were performed on DIV 7. The animals obtained from Weitong Lihua Experimentary Animal Central (Beijing, China). Before to remove the fetuses, the pregnant rats were placed in a euthanasia chamber (40 × 18.5 × 25 cm, Rurui Science and Technology Instruments Co., Ltd, Guangzhou, China), and then filled with 100% CO_2_ at a delivered rate of 0.2 L/s from a compressed gas cylinder for 3 min. Heart, breath, pupil and pain were detected to make sure the animal’s death before dissection. All animal experimental procedures were approved by the Animal Care Committee of Yunnan University.

### Experimental treatments

For the assessment of cell viability, cells were plated onto 96-well culture plates at 1 × 10^5^ cells per well. For western blot and PCR assays, cells were plated onto 6-well culture plates at 1 × 10^6^ cells per well. To investigate the toxicity effects of IL-1β, cells were treated at various times and with different concentrations of IL-1β, and cell viability was measured by MTT assay. To study the protective effects of EPA against IL-1β, the cells were pretreated with EPA for 40 min and then exposed to IL-1β for another 48 h. To determine whether or not the Akt/CREB signaling pathway was involved in the cell protection and BDNF regulation mediated by EPA, cells were pretreated with an Akt inhibitor (KRX-0401) for 30 min in conjunction with EPA and IL-1β. All experiments were performed using 3 separate cultures to confirm reproducibility.

### MTT assay

Cell viability was assessed by the MTT assay at a designated time after the various treatments. MTT was added to the culture media at a final concentration of 0.5 mg/mL, after 4 h incubation at 37 °C, the supernatant was removed, and the MTT formazan crystals were dissolved with dimethyl sulfoxide (DMSO), the absorbance at 578 nm was read using a scanning multiwell plate reader (Tecan, Switzerland) after shaking the plates.

### Western blot analysis

After the treatments, the cells were washed twice with cold phosphate-buffered saline (PBS) and harvested using cell scraper, and then lysed in RIPA buffer containing protease inhibitor mixture and phosphatase inhibitors on ice for 30 min. The protein concentrations were measured by BCA Protein Assay Kit. 30 µg of protein was separated by SDS polyacrylamide gel. Proteins were transferred to a PVDF membrane, incubated with fat-free milk for 2 h at room temperature for blocking nonspecific binding sites and further incubated overnight at 4 °C in fat-free milk containing the following primary antibodies: anti-Akt (1:1000), anti-phospho-Akt (1:1000), anti-CREB (1:2000), anti-phospho-CREB (1:1000), anti-BDNF (1:1000) and anti-GAPDH (1:5000). After washing, horseradish peroxidase (HRP)-conjugated secondary antibodies (1:5000) were applied to the membranes for 2 h at room temperature, immunostained bands were detected by enhanced chemiluminescence kit and quantified using Image-Pro Express 4.0 software (Media Cybernetics Inc. Rockville, MD, USA).

### Real-time PCR

The total RNA will be extracted from cultured hippocampal neurons using TRIzol reagent, and the first stranded cDNA synthesized from 1 µg total RNA using the Ominiscript RT kit according to the manufacturer’s instructions. Polymerase chain reactions was performed by the Corbett Life Science Rotor-Gene (Sydney, Australia) 6000 system,the PCR reaction solution contained 7.5 µL SYBR Green Mix, 0.5 µL each primer (10 µM), 1 µL cDNA and 5.5 µL RNase-free water. Cycling parameters were set at an initial 15 min step at 95 °C, followed by 45 cycles of 94 °C for 15 s, 59 °C for 30 s and 72 °C for 30 s. All reactions were performed in triplicate. Gene expression levels were normalized to the expression of reference gene (beta actin) with the ΔΔCt method [13]. The specific primer pairs (Shanghai SangonBiotech, Shanghai, China) were as follows: BDNF: 5′-CAAAAGGCCAACTGAAGC-3′ (forward) and 5′-CGCCAGCCAATTCTCTTT-3′ (reverse); β-actin: 5′-GTCGTACCA CTGGCATTGTG-3′ (forward) and 5′-CTCTCAGCTGTGGTGGTGAA-3′ (reverse).

### Statistical analysis

The data are presented as the mean ± SEM and statistical significance was evaluated by two-way analysis of variance (ANOVA) followed by the Bonferroni post hoc test using SPSS 18.0 software, with *P* values less than 0.05 considered to be statistically significant.

## Results

### Cell viability in IL-1β-incubated hippocampal neurons

**Fig. 1:**
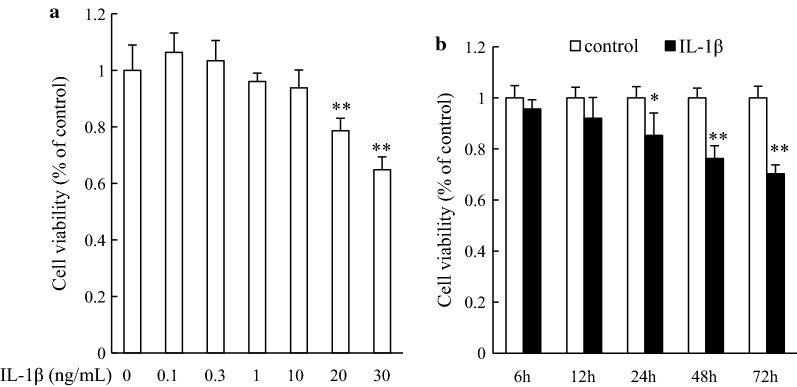
Cell viability was determined by MTT assay. **a** Cultured rat hippocampal neurons were treated with the indicated concentrations (0.1–30 ng/mL) of IL-1β for 48 h. **b** Cultured rat hippocampal neurons were treated with 20 ng/mL IL-1β for the indicated time. Percentage of cell viability was relative to the untreated control cells. *P < 0.05, **P < 0.01 versus control group (*n* = 6)

To determine the toxicity induced by IL-1β, we exposed the cultured hippocampal neurons to IL-1β (0.1–30 ng/mL) for 48 h, and the cell viability was assessed by the MTT assay. At the low IL-1β level (0.1 and 0.3 ng/mL), cell viability increased slightly, but there was no statistical difference compared to control cells (Fig. 1a). From 1 to 30 ng/mL, IL-1β induced cell damage in a dose-dependent manner, but only high concentration (20 and 30 ng/mL) IL-1β induced significant cell damage (both *P* < 0.01 compared to control cells), and the 30 ng/mL IL-1β elicited worse cell damage, which shown cell viability decreased sharply close to 60% of the control level. We then examined the time-dependent effect of IL-1β on cell damage. Figure 1b shows that cell viability was significantly decreased after hippocampal neurons were exposed to IL-1β for 24 h. Although cell viability showed more decline while the cells were exposed to IL-1β for 72 h, there was no significant difference compared to the cells exposed to IL-1β for 48 h. Based on this result, 20 ng/mL IL-1β and 48 h exposure time were selected for the subsequent experiments.

### EPA reversed IL-1β-induced cell damage, but the protective effect was blocked by inhibiting Akt

**Fig. 2:**
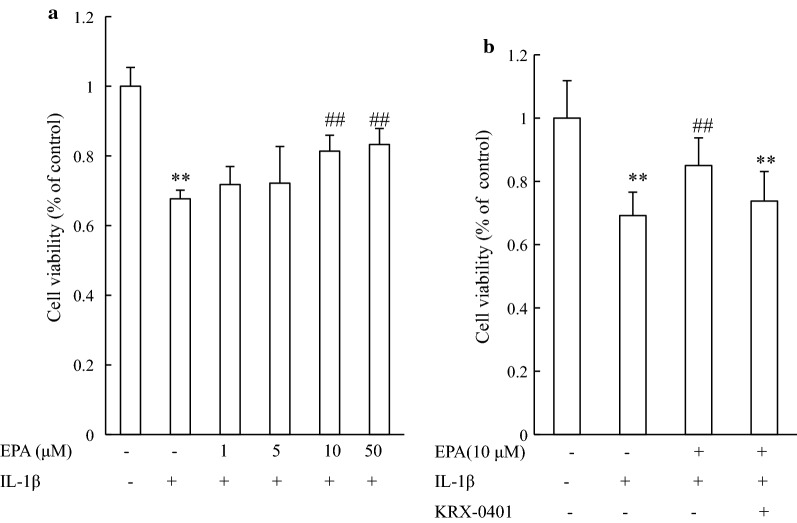
Protective effects of EPA on IL-1β triggered cell damage in cultured rat hippocampal neurons. Cell viability was determined by MTT assay. **a** Cells were pre-treated with the indicated concentrations (1–50 µM) of EPA for 40 min and then exposed to IL-1β (20 ng/mL) for another 48 h. **b** Cells were pretreated with KRX-0401 and EPA and then treated with IL-1β for 48 h. Percentage of cell viability was relative to the untreated control cells. **P < 0.01 versus control group; ##*P* < 0.01 versus IL-1β group

We then investigated the effects of EPA on IL-1β-induced cell damage. The MTT assay showed that pretreatment with EPA enhanced cell viability in a concentration-dependent manner (Fig. 2a). The pro-survival effect of EPA was observed at 10 µM (*P* < 0.01 compared to IL-1β treated cells). We next investigated if Akt signaling is involved in EPA’s neuroprotective effect. Hippocampal neurons were pretreated with KRX-0401 (45 µM) to inhibit Akt [22] and then exposed to IL-1β in the presence or absence of EPA (10 µM). Figure 2b shows that IL-1β triggered a significant decrease in cell viability, whereas EPA significantly alleviated cytotoxicity mediated by IL-1β; however, the protective effect of EPA against IL-1β-induced cell damage was attenuated by KRX-0401, suggesting the involvement of the Akt pathways.

### EPA rescued decline of Akt and CREB phosphorylation in IL-1β-treated hippocampal neurons, and this effect was blocked by Akt inhibitor

**Fig. 3:**
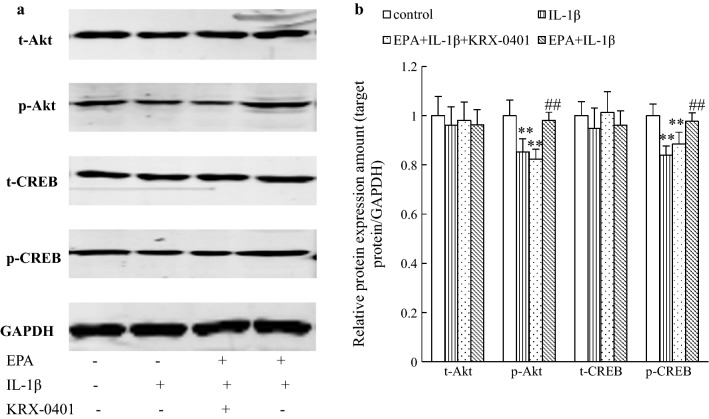
The effect of EPA on Akt and CREB phosphorylation was blocked by inhibition of the Akt signal, in the presence of IL-1β in cultured rat hippocampal neurons. **a** Cells pretreated with KRX-0401 and then treated with EPA and IL-1β, and the proteins expression was measured by western blotting. **b** Relative levels of proteins were determined by densitometry of the immunoblots. Data were normalized by taking the value of the control group as 1. **P < 0.01 versus control group; ##*P* < 0.01 versus IL-1β group

We assessed the role of the Akt/CREB pathway in the survival-promoting effect of EPA in hippocampal neurons. As shown in Fig. 3, IL-1β inhibited the phosphorylation of Akt and CREB in hippocampal neurons, which was consistent with the finding that IL-1β decreased cell viability. While the cells were cultured with EPA, the inhibitory effect of IL-1β on protein phosphorylation was reversed, which was also consistent with the results that the effect of EPA against cell damage was induced by IL-1β. However, when the cells were pretreated with KRX-0401 and then treated with EPA and IL-1β, the improvement of EPA on Akt and CREB phosphorylation and cell viability was blocked, confirming that the neuroprotective effect is mediated by the Akt/CREB pathway.

### EPA regulated BDNF levels via the Akt/CREB pathway

**Fig. 4:**
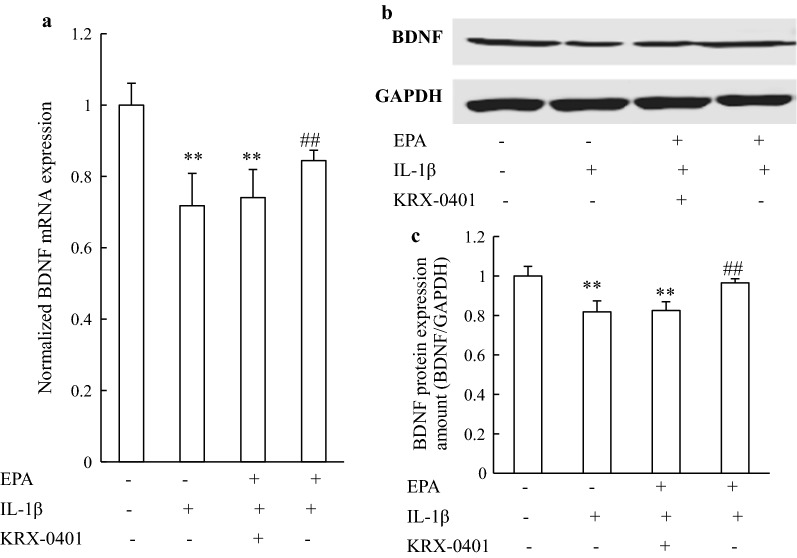
EPA reversed the inhibitory effects of IL-1β on the expression of BDNF via the Akt/CREB pathways in cultured rat hippocampal neurons. **a** BDNF mRNA and protein, **b** expression were measured by real-time PCR and western blotting, respectively. **c** Relative levels of BDNF protein were determined by densitometry of the immunoblots. Data from PCR and western blotting were normalized by taking the value of the control group as 1. **P < 0.01 versus control group; ##*P* < 0.01 versus IL-1β group

As shown in Fig. 4, IL-1β significantly down-regulated BDNF expression at both the mRNA and protein levels. The down-regulation of BDNF expression induced by IL-1β was also significantly attenuated by EPA. However, application of KRX-0401 inhibited the effects of EPA on BDNF expression, suggesting that EPA regulated BDNF expression in an Akt/CREB-dependent manner.

## Discussion

The present results showed that IL-1β caused neurotoxicity in hippocampal neurons, and EPA attenuated cell damage and BDNF down-regulation in correlation with Akt/CREB pathway activation. This notion is supported by the following observations: (1) Treatment with IL-1β in cultured hippocampal neurons caused cell damage, while EPA significantly reversed the toxic effect of IL-1β; (2) Akt and CREB phosphorylation was inhibited by IL-1β, while EPA prevented this inhibition; (3) inhibition of Akt/CREB blocked the neuroprotective effect of EPA toward IL-1β-induced cell damage; (4) the beneficial effect of EPA on BDNF expression was blocked by inhibiting Akt/CREB signaling. The present study confirms that EPA acts as a neuroprotector in the hippocampus and that this is accompanied by improving phosphorylation of Akt and CREB to augment BDNF expression.

IL-1β is a pluripotent proinflammatory cytokine that may activate a host’s defense system against infection and injury both in the peripheral immune system and in the central nervous system (CNS). Similar to Araujo and colleagues’ study, which reported that IL-1β is neurotoxic only at high concentrations and after relatively long exposure [23], in the present study, we found that low IL-1β (0.1 and 0.3 ng/mL) promoted cell growth slightly instead of exacerbating neuron damage. The beneficial effect of IL-1β on cell viability maybe due to IL-1β functioning as a paracrine growth factor to promote cell proliferation [24]. Indeed, our previous data reported that acute IL-1β administration can up-regulate nerve growth factor (NGF) expression, the increase in NGF levels could contribute to improving neuronal vitality [25]. Taking our findings together, we speculated that physiologically, even a slight increase in IL-1β levels benefit neuron health and regulate the expression of neurotrophic factors that play a role in this effect. In particular, the interrelations between low IL-1β and neurotrophic factor should also be investigated in the future.

Under abnormal conditions, such as in neurodegenerative disease, microglia can be over-activated in the CNS, and the over-activation of microglia can produce excessive proinflammatory cytokines, especially IL-1β [26]. Elevated IL-1β could cause a state of chronic inflammation and evoke multiple signaling, such as NF-κB and MAPK pathways, leading to cell damage [27–29]. Thus, we are not surprised to find that cell viability decreased significantly in the neurons that received high IL-1β in this study. More importantly, when the cells were preincubated with EPA, the cell viability was improved. This finding supports that EPA is highly neuroprotective against IL-1β. It should be pointed out that IL-1β can affect signal transduction associated with BDNF. For instance, it is has been reported that IL-1β suppressed Akt activation, the upstream signaling pathway involved in BDNF expression [30], it also demonstrated that IL-1β compromised phosphorylation of CREB, a transcription factor regulating BDNF expression [31], suggesting IL-1β places neurons at risk by interfering with BDNF signaling involving a Akt/CREB-associated mechanism. Our past work found EPA treatment restored BDNF decline induced by IL-1β administration rats provides a plausible explanation for the EPA’s neuroprotective effects maybe BDNF dependent [13]. Furthermore, the present findings showed that the protective effect of EPA against IL-1β-induced cell damage was attenuated by KRX-0401, it should be surmised that Akt/CREB may be a candidate to contribute the BDNF regulation mediated by EPA.

Akt is a member of the AGC (cAMP-dependent, cGMP-dependent and protein kinase C) kinase family. Activation of Akt via phosphorylation involved in multiple physiological and pathological effects response by phosphorylate a variety downstream molecules including of CREB [32–35]. Reportedly, the decrease in Akt and CREB phosphorylation was associated with neuron loss in neurodegenerative disease animal models, whereas enhanced Akt/CREB activity in the brain produced a beneficial effect on inhibiting neuroinflammation and improving neuronal function [36]. Under our experimental conditions, IL-1β decreased the phosphorylation of Akt and CREB. This is consistent with the finding of Soiampornkul et al. [37], who observed that the phosphorylation of Akt and CREB has been inhibited in cortical neurons that received IL-1β. Additionally, in our study, EPA antagonized the decline in Akt and CREB phosphorylation induced by IL-1β. However, in the presence of an Akt inhibitor, the EPA-mediated increase in Akt and CREB phosphorylation was partially blocked, as well as the EPA-dependent protective effect on neurons. Collectively, our data indicated that EPA may neutralize neurotoxicity mediated by IL-1β via Akt/CREB phosphorylation.

As stated above, BDNF is a direct target of CREB, and phosphorylated CREB binds to the cAMP response element (CRE) sequence 5′-TGACGTCA-3′ in the BDNF promoter region, this binding may promote the transcription activity of BDNF and increase its expression [38]. Consistent with the changes in CREB, we found that IL-1β suppresses CREB phosphorylation accompanied by a low BDNF expression, while EPA acted against these alterations. The deficiency of BDNF is believed to be a cause of neurodegeneration, and the up-regulation of BDNF may reduce neuroinflammation and hippocampal damage [39, 40]. Therefore, with the elevation of BDNF, the present results indicated EPA exposure restored cell viability that was inhibited by IL-1β; however, the application of an Akt inhibitor is sufficient to attenuate the positive effect of EPA on BDNF expression. Thus, it can be suggested that the regulation of BDNF by EPA under neuroinflammatory conditions is mediated by the Akt/CREB signaling pathway.

## Conclusions

Our findings extend previous data on the role of EPA in neuronal protection and show that EPA promoted the expression of BDNF, probably via activation of the Akt/CREB pathway. These results confirmed that EPA might constitute a promising alternative for the prevention of neuroinflammation-associated neurodegenerative diseases.

## Abbreviations

AD: Alzheimer’s disease Parkinson’s disease (PD)
AGC: cAMP-dependent, cGMP-dependent and protein kinase C
BDNF: brain-derived neurotrophic factor
CNS: central nervous system
CRE: cAMP response element
CREB: cAMP-response element binding protein
DMSO: dimethyl sulfoxide
EPA: eicosapentaenoic acid
IL-1β: interleukin-1β
MTT: methyl thiazolyl tetrazolium
NGF: nerve growth factor
PBS: phosphate-buffered saline
PD: Parkinson’s disease
PKA: protein kinase A
NF-α: tumor necrosis factor-alpha
